# Proton Pump Inhibitors in IPF: A Call for Clinical Trials

**DOI:** 10.3389/fphar.2018.00499

**Published:** 2018-05-17

**Authors:** Yohannes T. Ghebre

**Affiliations:** ^1^Department of Radiation Oncology, Baylor College of Medicine, Houston, TX, United States; ^2^Section of Pulmonary and Critical Care Medicine, Department of Medicine, Baylor College of Medicine, Houston, TX, United States

**Keywords:** proton pump inhibitors, antacids, generic drugs, clinical trials, IPF, inflammation, fibrosis

## Abstract

The recent FDA approval of two drugs, pirfenidone and nintedanib, for the treatment of idiopathic pulmonary fibrosis (IPF) has fueled interest in the development of additional drugs to treat the disease or its major clinical complications including cough and acute exacerbations. Since 2015, there are at least a dozen active interventional studies that are testing the efficacy of novel pharmacotherapies, exercise or stem cells in modifying the disease process in IPF. Additionally, there are combinatorial studies evaluating the effectiveness of pirfenidone or nintedanib in combination with other agents. However, there remains an urgent need for clinical trials to prospectively evaluate the efficacy of existing drugs with promising retrospective data, such as proton pump inhibitors (PPIs), in IPF. Several retrospective cohorts have provided tantalizing data supporting the beneficial effect of PPIs in patients with well-defined IPF. This review provides the general outlook of pharmacotherapies in IPF, and highlights preclinical and retrospective clinical data to make a case for randomized controlled clinical trials of PPIs in IPF.

## Introduction

The efficacy of pirfenidone and nintedanib in reducing disease progression including better preservation of lung function, exercise tolerance and progression-free survival has led to concurrent FDA approval of the two drugs for IPF (King et al., 2014; Richeldi et al., 2014). Although the clinical trials that led to the approval of these drugs reported manageable toxicity, the real world use of these drugs is additionally hampered by financial toxicity in that the cost of these drugs is unsustainably high (about $100,000 per patient per year). In addition, there is no guarantee that the cost of combining pirfenidone with nintedanib or that of new chemical entities (NCEs)-in-development for IPF would be any cheaper. In this regard, the pulmonary community has the opportunity to de-risk the process by encouraging the testing and development of existing FDA-approved drugs with demonstrated potential to favorably modify the disease process in IPF. The repurposing of such drugs has enormous potential to combat the soaring drug prices, increase access to standard of care, encourage patient compliance and minimize overall healthcare spending. This review article discusses preclinical and retrospective clinical data on the efficacy of PPIs, to stimulate interest in prospectively evaluating the therapeutic efficacy of these drugs in IPF. In addition, the general premise of pharmacotherapies in IPF is highlighted.

## Preclinical Evidence of PPIs as Anti-Inflammatory/Antifibrotic Drugs

Commonly known as “antacids,” PPIs have been in the market for over 20 years to treat or relieve several gastrointestinal disorders including inflammatory bowel disease, Barrett’s esophagus, Zollinger–Ellison syndrome and gastroesophageal reflux disease (GERD). Omeprazole (Prilosec), esomeprazole (Nexium), lansoprazole (Prevacid), and rabeprazole (Aciphex) are among the most commonly used PPIs. Several lines of preclinical research indicate that PPIs possess biological activities that extend beyond gastric acid suppression (**Table 1**). Some of the emerging effects of PPIs include regulation of oxidative stress through scavenging reactive oxygen species (ROS) and activation of endogenous antioxidant mechanisms including heme oxygenase 1 (HO1) (Suzuki et al., 1995, 1996; Zedtwitz-Liebenstein et al., 2002; Becker et al., 2006; Takagi et al., 2009; Ghebremariam et al., 2015). The later finding is particularly important given the reduced expression of HO1 and its downstream effector molecules in bronchoalveolar lavage samples obtained from IPF patients (Ye et al., 2008). Consistent with restoration of antioxidant defense mechanisms, PPIs have been shown to inhibit overall oxidation of proteins and lipids (Biswas et al., 2003). In addition, PPIs have been reported to block stress-induced DNA fragmentation and cellular apoptosis (Biswas et al., 2003). Similarly, we have demonstrated PPI-mediated inhibition of surfactant protein C (SP-C) positive lung epithelial cell apoptosis (Ghebremariam et al., 2015). Furthermore, PPIs have been demonstrated to exert potent anti-inflammatory action through inhibition of neutrophil infiltration and abrogation of classic pro-inflammatory molecules including tumor necrosis factor alpha (TNFα), interleukins (ILs), nuclear factor kappa-light-chain-enhancer of activated B cells (NFkB), intercellular adhesion molecule 1 (ICAM1), vascular cell adhesion molecule 1 (VCAM1) and inducible nitric oxide synthase (iNOS) (Wandall, 1992; Agastya et al., 2000; Yoshida et al., 2000; Handa et al., 2006; Kedika et al., 2009; Ghebremariam et al., 2015; Ghebre and Raghu, 2016b). Recently, it has also been reported that PPIs dose-dependently inhibit the gene expression of profibrotic markers such as collagen 1 (COL1A1), fibronectin 1 (FN1), matrix metalloproteinase 7 (MMP7), as well as proliferation of lung fibroblasts, transforming growth factor beta (TGFβ)-induced release of collagen, and cotton-smoke or bleomycin-induced lung inflammation and fibrosis (Ghebremariam et al., 2015; Nelson et al., 2017). The antifibrotic effect of PPIs in models of lung injury was reproduced in a model of carbon tetrachloride (CCl_4_)-induced liver fibrosis (Eltahir and Nazmy, 2018). Based on these novel observations, the idea of PPIs as antifibrotic agents has recently been surfaced (Ghebre and Raghu, 2016b). Overall, aggregated molecular, cell biological and preclinical data demonstrating favorable effect of PPIs on processes involved in lung oxidative stress, inflammation and fibrosis supports the beneficial effects of these drugs in measures of lung function and longevity in IPF patients. Therefore, translational studies need to evaluate the efficacy of combining PPIs with pirfenidone or nintedanib in comparison to the antifibrotic drugs alone. Such studies may be able to provide important insights into whether PPIs are likely to enhance the antifibrotic action of the current drugs in IPF and to establish basis for combinatorial clinical trials.

**Table 1:** Summary of proposed molecular mechanisms by which proton pump inhibitors (PPIs) regulate inflammation, oxidative stress, and fibrosis.

Proton pump inhibitor effect	Mechanism(s) of action	Reference
Anti-inflammatory	Downregulation of inflammatory molecules (e.g., TNFα, interleukins, adhesion molecules) by neutrophils, monocytes, endothelial and airway epithelial cells	Yoshida et al., 2000; Handa et al., 2006; Ghebremariam et al., 2015; Nelson et al., 2017
	Inhibition of neutrophil chemotaxis, degranulation and free radical production by polymorphonuclear neutrophils (PMNs)	Wandall, 1992; Suzuki et al., 1996
	Reduction of circulating inflammatory cells (e.g., monocytes)	Ohara and Arakawa, 1999
	Depletion of intracellular and extracellular neutrophil reactive oxygen species (ROS)	Zedtwitz-Liebenstein et al., 2002
	Downregulation of integrins such as CD11b (integrin α_M_β_2_) and CD18 (integrin β_2_)	Yoshida et al., 2000; Sasaki et al., 2005
	Upregulation of heme oxygenase 1 (HO1)	Becker et al., 2006; Takagi et al., 2009; Ghebremariam et al., 2015
	Downregulation of inducible nitric oxide synthase (iNOS)	Ghebremariam et al., 2015
Antioxidant	Direct ROS scavenging	Lapenna et al., 1996; Biswas et al., 2003; Simon et al., 2006
	Upregulation of HO1 and superoxide dismutase (SOD)	Becker et al., 2006; Namazi and Jowkar, 2008; Takagi et al., 2009; Ghebremariam et al., 2015
	Restoration of detoxifying enzymes such as glutathione (GSH), and improved mitochondrial function	Pastoris et al., 2008
Antifibrotic	Upregulation of HO1	Becker et al., 2006; Namazi and Jowkar, 2008; Takagi et al., 2009; Ghebremariam et al., 2015
	Inhibition of dimethylarginine dimethylaminohydrolase (DDAH) enzymatic activity	Ghebremariam et al., 2013, 2015; Ghebre and Raghu, 2016a
	Inhibition of TGFβ machinery, fibronectin and matrix metalloproteinase 7 (MMP7)	Ghebremariam et al., 2015
	Direct inhibition of fibroblast proliferation	Ghebremariam et al., 2015

*TNFα = tumor necrosis factor alpha, TGFβ = transforming growth factor beta.*

## Proton Pump Inhibitors (PPIs) in IPF: Clinical Evidence

In IPF, the strong association of the disease with GERD has justified the frequent prescription of PPIs to IPF patients. Unfortunately, despite maintaining or escalating PPI therapy, IPF patients continue to experience abnormal gastroesophageal reflux (GER) including hypersecretion of gastric juice and/or clinical symptoms such as heartburn, nausea, chest pain and regurgitation (Tobin et al., 1998; Raghu et al., 2006a; Ghebremariam et al., 2015). In addition, the disease process in IPF may be aggravated by potential reflux and microaspiration of gastric contents into the lungs (Patti et al., 2005; Salvioli et al., 2006; Sweet et al., 2007). Paradoxically, the PPIs, while unable to stop reflux or microaspiration, have been associated with profound beneficial outcomes in IPF including fewer episodes of acute exacerbations, slower decline in lung function, prolonged transplant-free survival, reduced all-cause and IPF-related mortality (Raghu et al., 2006b; Lee et al., 2011, 2013, 2016; Noth et al., 2012; Ghebremariam et al., 2015; Ghebre, 2016). In 2005, Raghu et al. reported that the use of PPIs, without conventional IPF therapy, was associated with stabilization of lung function (Raghu et al., 2006b). In addition, there were no episodes of acute exacerbations or respiratory-related hospitalizations among the IPF patients taking PPIs. By contrast, poor PPI adherence was correlated with deteriorations of lung function. This novel observation provokes important questions including how the use of PPIs was associated with stabilized or improved lung function in IPF patients whose conditions would otherwise have deteriorated, why poor PPI adherence contributed to loss of lung function, and how PPI use was associated with the absence of acute exacerbations or any hospitalization for respiratory-related illnesses for up to the 6 years of follow-up time in IPF patients who otherwise refused to receive the standard of care therapy. This curiosity was further elaborated in 2011 when Lee et al. (2011) published stimulating data gathered from interstitial lung disease (ILD) databases of two geographically distinct institutions – the University of California, San Francisco and Mayo Clinic. In this cohort of 203 IPF patients, the use of PPIs/antacids was associated with significantly lower lung fibrosis score at baseline and prolonged survival time compared to IPF patients that did not receive the medication. In fact, the use of “antacid therapy” was an independent predictor of longevity in this cohort. Given that about 87% of the patients classified as “taking GER medications” were on PPIs, the use of PPIs is chiefly responsible for the 2.2-fold greater transplant-free survival time in this group compared to the group that did not receive the medication (median survival time of 5.4 years vs. 2.5 years in the control group; *p* < 0.01). Accordingly, the hazard ratio (HR) in the treatment arm was intriguingly reduced to 0.5 (Lee et al., 2011). In 2012, Noth et al. (2012) from the University of Chicago reported that IPF patients on “anti-reflux therapy” (95% were on PPIs) had significantly better lung function (as shown by greater diffusing capacity for carbon monoxide; DL_CO_) and reduced composite physiologic index (CPI); a validated measure of disease severity in IPF (Wells et al., 2003). Surprisingly, this observation was true in the absence of a direct correlation between the presence of hiatal hernia and severity of lung function (Noth et al., 2012). The presence of GER/GERD and hiatal hernia are often described as orchestrators of the disease process in IPF (Tobin et al., 1998; Linden et al., 2006; Raghu et al., 2006a; Hoppo et al., 2011).

**FIGURE 1:**
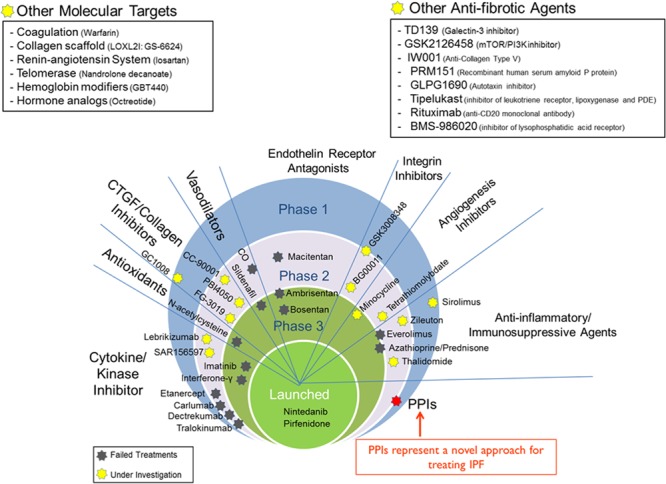
Drug development in IPF. Candidate drugs are pinned down at the clinical stage they currently are or have failed at.

In 2013, the IPF Clinical Research Network (IPFnet) group analyzed three ILD databases containing 242 patients who participated in three large randomized controlled trials (STEP-IPF, ACE-IPF, and PANTHER-IPF) (Lee et al., 2013). Although the drugs primarily studied in these clinical trials (sildenafil, warfarin and the “triple therapy” of prednisone, azathioprine and *N*-acetylcysteine) have failed to meet their endpoints (**Figure 1**), the use of PPIs among the patients who were designated in the “placebo” arm of these 3 studies was significantly associated with lower loss in lung function (as shown by lower loss in forced vital capacity; FVC) and fewer acute exacerbations. In addition, there was a trend toward fewer all-cause hospitalizations and all-cause mortality in the patients who received GER medications (over 90% of whom on PPIs) compared to these without the medications.

In 2015, we have reported data analyzed from the ILD databases of two academic hospitals – Stanford University and University of Alabama at Birmingham (Ghebremariam et al., 2015). In this study of 215 IPF patients of whom 130 were on PPIs, we found that the use of PPIs as “add on” therapy was associated with longer transplant-free survival time (median survival time of 3.4 years vs. 2 years in the control group; *p* < 0.01) compared to these who were only on standard of care. In a subgroup analysis of IPF patients with no symptoms of GERD, the use of PPIs was also associated with significantly longer survival time (*p* = 0.009) (Ghebremariam et al., 2015). In the same year, Lee et al. (2016) analyzed data from 786 IPF patients in their ILD database at Seoul National University in South Korea and found that the duration of PPI use was progressively associated with lower IPF-related mortality in that PPI use for over 4 months provided greater survival time compared to use of the medication for 2 or 3 months. Intriguingly, their univariate and multivariate Cox regression analysis shows that the duration of PPI use but not diagnosis of GERD was significantly associated with lower IPF-related mortality.

## Proton Pump Inhibitors (PPIs) in the Era of Pirfenidone and Nintedanib

The curiosity of documented beneficial outcomes associated with the use of PPIs has led to querying the data gathered from the INPULSIS (nintedanib) (Richeldi et al., 2014), as well as CAPACITY and ASCEND (pirfenidone) trials (King et al., 2014) in order to address the effect of antacids on disease outcome in IPF. *Post hoc* analysis of the INPULSIS data comparing 1061 IPF patients treated with antacids (406 of these patients received PPIs or H2 receptor antagonists; H2RA) at baseline versus 655 patients who did not receive antacids at baseline. This dataset did not show any beneficial effect of antacids on lung function as demonstrated by lack of effect on the change in FVC (Raghu et al., 2015a). However, this study suffers from major limitations including the lack of information on whether the patients who received antacid medications at baseline continued on these medications, the possibility of cross-over where these who initially designated as “no antacid group” started antacid medications during the course of the study and vice versa. Notably, there were also about 40% more IPF patients in the “no antacid group” (*n* = 394) compared to the “antacid group” (*n* = 244). In other words, there were presumably more patients who were taking the antifibrotic drug nintedanib in the “no antacid group.” Thus, the beneficial effect of nintedanib is likely to influence the possible efficacy of antacids. In fairness, the data should have separated the placebo arm and the nintedanib arm and then compared the effect of antacid medications within the placebo arm and/or within the nintedanib arm.

The CAPACITY/ASCEND study also analyzed a database of 624 IPF patients who were randomized into the placebo arm of the pirfenidone study (Kreuter et al., 2016). In this study, there were equivalent number of patients in the “antacid therapy” group (*n* = 291) in comparison to the “no antacid therapy” group (*n* = 333). After adjustment for several confounders, this study showed positive trends favoring the antacid group (of whom about 90% were on PPIs) in terms of IPF-related mortality, death or 6-min walk distance (6MWD) decrease by 10% or more, progression-free survival and all-cause mortality (Ghebre, 2016; Kreuter et al., 2016). There was, however, an increased risk of nonfatal infection in the sickest quartile of the antacid group. It should, however, be noted that the findings of increased infection in the antacid group is based on unadjusted data.

A separate cohort comparing the pirfenidone arm of the CAPACITY/ASCEND trials between IPF patients who were on antacids plus pirfenidone (*n* = 273) versus pirfenidone alone (*n* = 350) found that the use of antacids (of whom about 90% received PPIs) on top of pirfenidone was associated with statistically significant reduction in the loss of lung function by 10% or more (≥10% FVC decrease) compared to pirfenidone alone (*p* = 0.0273) (Ghebre, 2016; Kreuter et al., 2017). Antacid “add on” was also numerically favored, without reaching statistical significance, in terms of IPF-related mortality, progression-free survival, death or 6MWD decrease by 10% or more, and all-cause mortality. In addition, the increased risk of overall infection in the first study (Kreuter et al., 2016) did not hold its significance in the second study (Kreuter et al., 2017). Similar to the limitations of the antacid data analyzed from the INPULSIS trial, the data in the CAPACITY/ASCEND analysis also suffers from lack of information about the continuation of the antacid therapy beyond baseline. In this regard, the data reported by the IPFnet group (Lee et al., 2013) has superiority in that over 90% of the IPF patients who used antacids at baseline were confirmed to have continued to take the medications for the duration of the 52-weeks study.

Overall, analysis of the retrospective clinical data gathered from over 3,500 IPF patients clearly implies beneficial outcomes associated with the use of antacid therapy in general and PPIs in particular. These promising datasets and the aggressive nature of the disease shoulders the IPF community an urgent task of prospectively evaluating PPIs in IPF. Such randomized controlled clinical trials have already started to emerge^1^ (NCT02085018). However, larger and longer duration studies are required to definitively address the value of PPIs in IPF. The conditional recommendation of PPIs for the treatment of IPF by the North American, European, Japanese and Latin American Thoracic Societies (Raghu et al., 2015b) amplifies the urgency of this overdue call for clinical trials.

## The Outlook of IPF Pharmacotherapy

The glimpse of hope ignited by the FDA-approval of two drugs for IPF is being tampered by the modest efficacy and unsustainably high cost of these drugs. In addition, the continued failure of drugs-in-development that ravaged the IPF field prior to the era of pirfenidone and nintedanib is thwarting the renewed optimism. The issue of high cost of drugs may be calibrated by drawing important lessons from the cancer field where over 100 prominent oncologists have objected spiraling prices of cancer drugs by urging re-evaluation and new regulation of drug costs (Kantarjian et al., 2014; Workman et al., 2017). In addition, supporting and expanding the testing and development of repurposed drugs, especially generic ones, may prove to be essential in circumventing the issue of financial toxicity and in transforming IPF-related medical expenses across the globe. However, it should be emphasized that it is not the intention of this article to suggest lower bar of approval for repurposed or cheaper drugs. These drugs should well be held against the highest standards of clinical trials and post-market pharmacovigilance in the IPF patient population; even when proven to be harmless in the general patient population for whom they were originally approved.

So far, a number of molecules and signaling pathways have been targeted in IPF. Some of these targets include cytokines, kinase enzymes, collagen scaffold, telomerase, phosphodiesterase, endothelin receptors, integrins, oxidative stress, coagulation, angiogenesis, inflammation, and the renin-angiotensin system. Despite the array of targets, the failure rate is daunting (**Figure 1**). Nonetheless, the widespread issue of negative clinical trials in IPF needs to shape the design and commencement of future clinical trials, as well as spark more innovative strategies to discover newer molecular targets and refine existing ones. In addition, there is a need to increase ancillary support for translational research aimed at performing high-throughput (HTS) and ultra-high-throughput (uHTS) drug screening of libraries composed of existing drugs and NCEs.

## Conclusion

Based on encouraging retrospective clinical data, the leading Thoracic Societies have recently recommended the use of “antacids” in IPF with the treatment indication being IPF and not GERD (Raghu et al., 2015b). However, this recommendation is based on retrospective observations and carries very low confidence in effect estimates. Therefore, conducting antacid therapy clinical trials are the need of the hour. There is an ongoing clinical study evaluating the effect of surgical correction of reflux on measures of lung function (WRAP-IPF; NCT01982968). This study, however, is addressing a different set of questions revolving around the role of GERD in IPF. The outcome of this study, positive or negative, should not deter testing of the hypothesis that antacids such as PPIs possess biological activity that extend above and beyond gastric acid suppression to favorably modify the disease process in IPF. In addition, any nonfatal side effects such as possible increases in the risk of respiratory infections upon PPI use should be dealt on a case-by-case basis rather than being used to monger skepticism and premature judgement.

## Author Contributions

YG conceived the idea and wrote the manuscript.

## Conflict of Interest Statement

YG is an inventor on patents, owned by Stanford University, that protect the use of agents, including proton pump inhibitors (PPIs), for therapeutic use of new indications including IPF.

## Footnotes

1 https://clinicaltrials.gov
